# Late-responding normal tissue cells benefit from high-precision radiotherapy with prolonged fraction delivery times via enhanced autophagy

**DOI:** 10.1038/srep09119

**Published:** 2015-03-13

**Authors:** Qiwei Yao, Rong Zheng, Guozhu Xie, Guixiang Liao, Shasha Du, Chen Ren, Rong Li, Xiaoshan Lin, Daokun Hu, Yawei Yuan

**Affiliations:** 1 Department of Radiation Oncology, Nanfang Hospital, Southern Medical University, Guangzhou, Guangdong 510515, P.R. China

## Abstract

High-precision radiotherapy (HPR) has established its important role in the treatment of tumors due to its precise dose distribution. Given its more complicated delivery process, HPR commonly requires more fraction delivery time (FDT). However, it is unknown whether it has an identical response of prolonged FDT on different normal tissues. Our results showed that fractionated irradiation with prolonged FDTs (15, 36, and 50 minutes) enhanced cell surviving fractions for normal tissue cells compared with irradiation with an FDT of 2 minutes. However, the late-responding normal cell line HEI-OC1 was more responsive to prolonged FDTs and demonstrated higher surviving fractions and significantly decreased apoptosis and DNA damage compared to the acute-responding normal cell line HaCaT. Increased autophagy mediated via the ATM-AMPK pathway was observed in HEI-OC1 cells compared with HaCaT cells when irradiated with prolonged FDTs. Furthermore, treatment with the autophagy inhibitor 3-MA or ATM inhibitor KU55933 resulted in enhanced ROS accumulation and attenuation of the effect of prolonged FDT-mediated protection on irradiated HEI-OC1 cells. Our results indicated that late-responding normal tissue cells benefitted more from prolonged FDTs compared with acute-responding tissue cells, which was mainly attributed to enhanced cytoprotective autophagy mediated via the ATM/AMPK signaling pathway.

Radiotherapy (RT) is an important therapeutic method for cancer treatment. The dose delivered to tumor tissue was limited due to the appearance of acute and late toxicities caused by radiation, such as mucositis, dysphagia, xerostomia, dermatitis, hearing loss and pulmonary fibrosis, which may have profound effects on the patient's life span and quality of life. High-precision radiotherapy (HPR), i.e., intensity-modulated radiation therapy (IMRT), has steadily established its role in cancer treatment. Increasing clinical data have shown the advantage of HPR due to its excellent dose distribution in the target volume and sparing of normal tissues^1^,^2^,^3^,^4^. Compared to conventional external beam radiotherapy (EBRT), HPR usually requires more fraction delivery time (FDT) due to the more complicated delivery process (approximately 1 to 3 minutes for conventional EBRT and 15 minutes or more for HPR, i.e., prospective respiratory gating Image-guided IMRT usually takes more than 50 minutes to deliver the same fraction dose).

Prolonged FDT has been demonstrated to reduce radiation-induced cell death in Chinese hamster fibroblast cells^5^. Acute- and late-responding normal tissues have different α/β ratios and usually generate a distinct biological response to different patterns of radiation^6^,^7^. However, it is unclear whether irradiation with prolonged FDTs produces an identical protective effect on acute- and late-responding normal tissue. If prolonged FDTs have different effects on radiation-induced acute- and late-responding normal tissue toxicities, the effects might affect the patient's quality of life. Thus, it is necessary to investigate the effect of prolonged FDTs on radiation toxicity in acute- and late-responding head and neck normal tissue.

Radiation-induced sensorineural hearing loss (SNHL) is a common, late, and permanent complication in cancer patients managed with radiotherapy. In addition, hair cell damage caused by irradiation of the inner cochlea is the major reason for SNHL^8^. Radiation-induced severe skin response and oral mucositis are common acute adverse effects of radiotherapy for cancer; they are thought to result from excessive inflammation and epithelial ablation, including keratinocyte damage caused by radiation therapy. Thus, we used HEI-OC1 hair cells as late-responding tissue cells and HaCaT human keratinocytes as acute-responding tissue cells to investigate the response of different normal tissues to irradiation with prolonged fraction delivery times (Fig. 1). Our results indicated that late-responding normal tissue cells benefitted more from prolonged FDTs compared with acute-responding tissue cells, which was mainly attributed to an enhanced cytoprotective autophagy mediated by the ATM/AMPK signaling pathway.

## Results

### Acute- and late-responding normal tissue cells exhibit differential sensitivity to prolonged fraction delivery times

Single-dose irradiations (0, 2, 4, 6, 8, 10 Gy) were performed to determine the α/β ratio for HEI-OC1 cells and HaCaT cells. The α/β ratio was approximately 1.096 and 9.83 for HEI-OC1 cells and HaCaT cells, respectively (Fig. 2). These results were consistent with previous reports indicating that the late-responding normal tissue cell line HEI-OC1 had a lower α/β ratio compared to the acute-responding normal tissue cell line HaCaT^7^.

To investigate the effect of different fraction delivery times on acute and late responding normal tissue cell survival, the clonogenic surviving fraction was measured in HEI-OC1 cells and HaCaT cells. As shown in Fig. 2, the surviving fractions after irradiation simulating HPR with a prolonged FDT of 15, 36, or 50 minutes were higher than those simulated using conventional EBRT, and exhibited statistical significance in both HEI-OC1 and HaCaT. Importantly, between the different irradiation protocols simulating HPR, there were also significant differences in cell survival for HEI-OC1 and a marginal significance for HaCaT (Supplementary Table S1 online).

To determine whether prolonged fraction delivery times affected the production and repair of DNA double-strand breaks (DSBs) induced by irradiation, cells irradiated with different FDTs were examined for radiation-induced foci (RIF) formation using γ-H2AX immunofluorescence^9^. Radiation-induced γ-H2AX foci were detectable at 6 and 30 hours after irradiation in all of the groups, and γ-H2AX foci were decreased at 30 hours after irradiation (Fig. 3a). Interestingly, compared to HaCaT cells, HEI-OC1 cells showed a significant decrease in γ-H2AX foci formation with prolonged FDT (Fig. 3b, c).

To further study the effect of prolonged fraction delivery time on apoptosis of HEI-OC1 and HaCaT cells, apoptosis of both cells was measured using flow cytometry with the Annexin V/PI apoptosis detection kit. As shown in Fig. 4, groups exposed to a prolonged FDT of 16, 37, and 51 minutes showed a decrease in the percentage of apoptotic cells compared to the group with a FDT of 2 minutes for both cells. However, this reduction in apoptosis was more obvious in HEI-OC1 cells compared to HaCaT cells (Fig. 4c). Taken together, these results suggested that the late-responding normal tissue cell line HEI-OC1 was more sensitive to radiation with prolonged FDTs than early-responding normal tissue cell line HaCaT.

To test whether this is representative of the majority of late and early responding tissues, we further determined the response to prolonged FDTs by colony formation assay using another two normal tissue cell lines with different α/β ratios, a human normal liver cell line HL-7702 and a mammary epithelial cell line MCF-10A. Cells were exposed to radiation with different FDTs. Consistent with above results, HL-7702 cells whose α/β ratio was 0.96 indeed benefited more from prolonged FDTs than MCF-10A cells whose α/β ratio was 12.39 (Supplementary Fig. S1 online). These results, to some extent, further support the phenomenon that late-responding normal tissue cells benefit more from HPR with prolonged FDTs.

### Prolonged fraction delivery time engages the ATM/AMPK signaling pathway to induce autophagy in HEI-OC1

To explore the mechanism underlying the different responses to prolonged FDTs between HEI-OC1 and HaCaT cells, we examined the autophagic activities in these two cell lines. LC3, p62 and Beclin1 proteins were analyzed to assess the effect of different irradiation protocols on the autophagic pathway^10^. Interestingly, for the late-responding tissue cell line HEI-OC1, irradiation induced a dramatic increase in the amount of LC3-II and Beclin1 and a decrease in the amount of p62. However, in the acute-responding tissue cell line HaCaT, there was only a slight change in autophagy related proteins following irradiation, as shown in Fig. 5a. In HEI-OC1 cells, prolonged FDT increased the levels of LC3-II and Beclin1 and decreased the level of p62 compared to a FDT of 2 minutes, while prolonged FDTs did not induce different autophagic activity in HaCaT cells (Fig. 5a). In addition, a GFP-LC3 assay was performed using a laser scanning fluorescence confocal microscope to measure autophagy. As shown in Fig. 5b and c, prolonged FDTs resulted in an enhanced accumulation of GFP-LC3 puncta in HEI-OC1 cells but not in HaCaT cells. To confirm that the increased autophagosome formation was due to increased autophagic flux, cells were treated with Bafilomycin A1 (BafA1), which blocks lysosomal degradation of autolysosome contents. Consistent with previous results, in the presence of 100 nM BafA1, exposure to IR with a prolonged FDT increased the level of LC3-II (Fig. 5d).

Ataxia-telangiectasia mutated (ATM) and AMP-activated protein kinase (AMPK) have been reported to activate and regulate autophagy in cells exposed to stress^11^,^12^. Thus, we investigated ATM and AMPK signaling in HEI-OC1 cells after irradiation. As shown in Fig. 5e, exposure of HEI-OC1 cells to IR with prolonged FDT activated ATM and AMPK signaling, and treatment with the ATM inhibitor KU55933 at a concentration of 100 nM decreased levels of P-ATM and P-AMPK. Interestingly, the enhanced autophagic activity induced by prolonged FDTs was attenuated after inhibition of the ATM signal. Taken together, these results suggested that prolonged FDTs engages the ATM/AMPK signaling pathway to induce enhanced autophagic activity in HEI-OC1 cells, but not in HaCaT cells.

### Autophagy contributes to the protective effect induced by prolonged FDT on HEI-OC1 cells via ROS deletion

To investigate the relationship of increased autophagy caused by prolonged FDTs in HEI-OC1 and the enhanced sensitivity of late-responding normal tissue cell to prolonged FDTs, we examined whether modulation of the autophagic pathway affected cell survival in cells treated with different irradiation protocols. We analyzed the effects of the autophagy inhibitor 3-MA and ATM inhibitor KU55933 on clonogenic survival in different groups. For cells pre-treated with 3-MA at a concentration of 5 mM for 1 h before irradiation, the level of LC3-II significantly decreased, which indicated an inhibition of autophagy (Fig. 6a). Exposure to 3-MA or ATM inhibitor KU55933 did not significantly change the colony number in the control group of both two cell lines (Supplementary Fig. S3 online). As shown in Fig. 6b, HEI-OC1 cells demonstrated significantly more colony numbers when irradiated with prolonged FDT for 16, 37, and 51 minutes compared with a FDT of 2 minutes. After treatment with 3-MA or KU55933, a decrease in colony numbers was found compared to irradiation alone in all groups, and this decrease was more significant when combined with prolonged FDT. These results indicated that the inhibition of autophagy may exacerbate HEI-OC1 cells' radiation damage and this effect was more obvious when irradiated with prolonged FDT.

Irradiation induces ROS generation, which results in DNA damage, and ROS accumulation has been shown to be regulated by autophagy^13^,^14^. Thus, it is possible that the elevated autophagy induced by prolonged FDTs in irradiated HEI-OC1 cells may also serve as an adaptation to prevent the accumulation of ROS. Thus, we detected the intracellular ROS level using fluorescent probe dichlorofluorescin diacetate (DCFH-DA). Irradiation increased ROS levels in HEI-OC1 cells, and when exposed to radiation with prolonged FDT, the intracellular ROS level decreased. After treatment with 3-MA or KU55933, the intracellular ROS level increased and the reduction in the ROS level induced by prolonged FDTs disappeared (Fig. 6c). These results indicated that autophagy induced by prolonged FDTs protects HEI-OC1 cells from irradiation injury via a decrease in ROS.

## Discussion

In this study, our results indicated that although prolonged FDTs simulating HPR increased cell surviving fractions for both acute-responding and late-responding normal tissue cell lines, late-responding normal tissue cell lines obtained greater benefit from prolonged FDTs.

Clinical and experimental results showed a distinct response to radiation in acute- and late-responding normal tissues due to their different characteristics. Late-responding normal tissue with a lower α/β ratio showed a stronger ability to demonstrate sub-lethal damage repair (SLDR) compared to acute-responding normal tissue. When the fraction delivery time was prolonged, SLDR occurred during the inter-subfraction^6^,^15^. Our results revealed that acute- and late-responding normal tissues have different sensitivities to changes in FDT (Fig. 2). Olive et al. showed that DNA repair might underlie the increase in survival for tumor cells when the dose delivery is prolonged^16^. Our data suggested that for normal tissue cells, particularly for late-responding tissue cells, prolonged FDTs decreased radiation-induced DNA damage due to enhanced DNA repair during the prolonged fraction delivery process.

Autophagy represents a dynamic lysosomal pathway responsible for degrading organelles and long-lived proteins. In addition, it is important in maintaining intracellular homeostasis and cell health, which can be activated as an adaptive response to adverse environmental conditions, such as deprivation of nutrients, hypoxia and different types of therapeutic stress. Furthermore, autophagy is expected to be cytotoxic during irradiation. However, many laboratories have recently found that autophagy is frequently activated in radio-resistant cancer cells, where it elicits a cell survival strategy^17^. Hou et al. showed that autophagy may play an important role in protecting stemness of mesenchymal stem cells from irradiation injury^14^. Thus, it was suggested that autophagy may protect cells from radiation damage not only in cancer cells but also in some normal tissue. In our study, radiation-induced autophagic activity was enhanced only in HEI-OC1 cells when irradiated with prolonged FDT, and inhibition of autophagy decreased the protective effect of prolonged FDTs to a level that was similar to the effect observed in HaCaT cells (Fig. 6). Thus, it was suggested that autophagy might mediate the difference in sensitivity to prolonged FDTs between acute- and late-responding normal tissues.

The regulatory role of ATM in autophagy has been previously identified by other investigators. When exposed to genotoxic and oxidative stress, such as IR and ROS, ATM is activated and can regulate autophagy^12^,^18^. Our results showed that irradiation with prolonged FDT activated ATM signaling, which further stimulated AMPK signaling, resulting in the induction of autophagy. This elevated autophagy eliminated radiation-induced intracellular ROS accumulation. Radiation-induced ROS in HEI-OC1 cells has been reported to play a key role in the promotion of apoptosis by affecting mitochondrial permeability, release of cytochrome c, and activation of p53 and caspases^19^. Suppression of ROS accumulation, which could protect cells from radiation-induced cytotoxicity, inhibited radiation-induced apoptosis in HEI-OC1 cells^20^. Here, we found that the reduction of the apoptotic cells after irradiation with prolonged FDT was stronger in late-responding normal tissue cells (Fig. 4), and autophagy may contribute to this phenomenon via the elimination of radiation-induced ROS. Because autophagy plays multiple roles in regulating cell survival, the function of autophagy in normal tissue cells may be complicated. To decrease radiotherapy toxicity, additional studies are required to demonstrate the mechanism underlying normal tissue radiation damage.

Previous studies have shown that prolonged FDTs can cause a significant decrease in cell killing in tumor cells with a low α/β ratio, but only slight decrease, without significance, in tumor cells with a high α/β ratio^15^,^21^. We also irradiated nasopharyngeal carcinoma cell lines CNE1 and CNE2 with the same dosing schedules that had been used for normal cells. Compared to CNE2 with a α/β ratio of 10.66, CNE1 with a α/β ratio of 3.66 was more sensitive to radiation with prolonged FDTs (Supplementary Fig. S2 online). Since a large number of evidence supports that most of tumors in human body have a high α/β ratio^6^, the current precise RT pattern with prolonged FDTs actually does not significantly attenuate cell killing effects for most tumors. Notably, our results from normal tissue cell lines also showed that the prolonged FDTs, when compared with conventional EBRT, significantly increased cell protection in late-responding normal tissue cells with a low α/β ratio, and that only slight protection was observed in acute-responding normal tissue cells with a high α/β ratio. Based on these findings, we speculate that the current precise RT pattern with prolonged FDTs may not obviously benefit the patients whose tumors are surrounded by a lot of acute-responding normal tissues, because the prolonged FDTs contribute simultaneously to cell-surviving of both tumors and acute-responding normal tissues with the high α/β ratio slightly. However, the current precise RT pattern with prolonged FDTs would obviously benefit the patients whose tumors are surrounded by a lot of late-responding normal tissues, for example intracranial tumors, because the prolonged FDTs contribute to more cell-surviving of late-responding normal tissues with the low α/β ratio but not tumors that have a high α/β ratio. So when tumors are surrounded by a lot of late-responding normal tissues, increasing radiation does within the biologically tolerable does range of late-responding normal tissues may be an appropriate approach to further improve tumor therapeutic effect. Given that late-responding normal tissues may have high tolerance doses when receiving radiotherapy with prolonged FDTs, and current tolerance dose metrics of normal tissue were based on conventional radiotherapy, therefore, it would be meaningful to re-quantity the tolerance dose of late-responding normal tissues under the condition of HPR with prolonged FDTs, which may offer optimal dose for treatment planning of current HPR. Moreover, because radiation-induced normal tissue damage *in vivo* is more complicated than cell lines damage in vitro^22^, future studies should focus on *in vivo* experiments to confirm the effect of prolonged FDTs on radiation-induced toxicity in acute- and late-responding normal tissues.

In summary, this study showed that prolonged FDTs protect normal tissue cells from radiation damage, and these effects may be strengthened with more prolonged FDTs. Late-responding normal tissue demonstrated a greater benefit from changes in fraction delivery time compared to acute-responding tissue because prolonged FDTs induced a stronger cytoprotective autophagy by engaging the ATM/AMPK signaling pathway. The different effects of dose protraction for acute- and late-responding normal tissues, if confirmed by *in vivo* and clinical studies, may be considered during radiotherapy.

## Methods

### Cell lines and culture

HEI-OC1 immortalized mouse auditory hair cells were kindly provided by Professor F. Kalinec (House Ear Institute, Los Angeles, CA, USA)^23^. The cells were maintained in Dulbecco's modified Eagle's medium (DMEM) with 10% FBS at 33°C under 10% CO_2_ in air. HaCaT human keratinocyte cells (China Center for Type Culture Collection, CCTCC)^24^ were cultured in DMEM with 10% fetal bovine serum. Human normal liver cell line HL-7702 and Human nasopharyngeal carcinoma cells CNE1, CNE2 were obtained from the Cell Bank of Type Culture Collection of the Chinese Academy of Sciences (Shanghai, China) and cultured in RPMI1640 with 10% fetal bovine serum. Human mammary epithelial cell line MCF-10A were from American Type Culture Collection (ATCC) and cultured in DMEM/F12 (1:1) medium supplemented with 5% horse serum, 100 ng/ml cholera toxin, 20 ng/ml epidermal growth factor, 0.5 μg/ml hydrocortisone and 10 μg/ml insulin. All cells except HEI-OC1 were maintained in an incubator at 37°C under 5% CO2 in air.

### Irradiation

**Figure 1:**
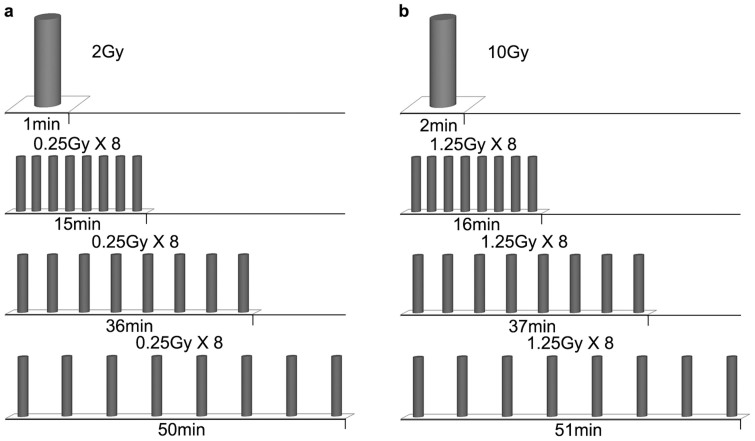
Radiation dose–delivery scheme. (a) 2 Gy and (b) 10 Gy were delivered continuously or intermittently with 8 subfractions with inter-subfraction intervals of 2, 5 and 7 minutes (total fraction delivery time of 15, 36 and 50 minutes for 2 Gy or 16, 37, and 51 minutes for 10 Gy).

**Figure 2:**
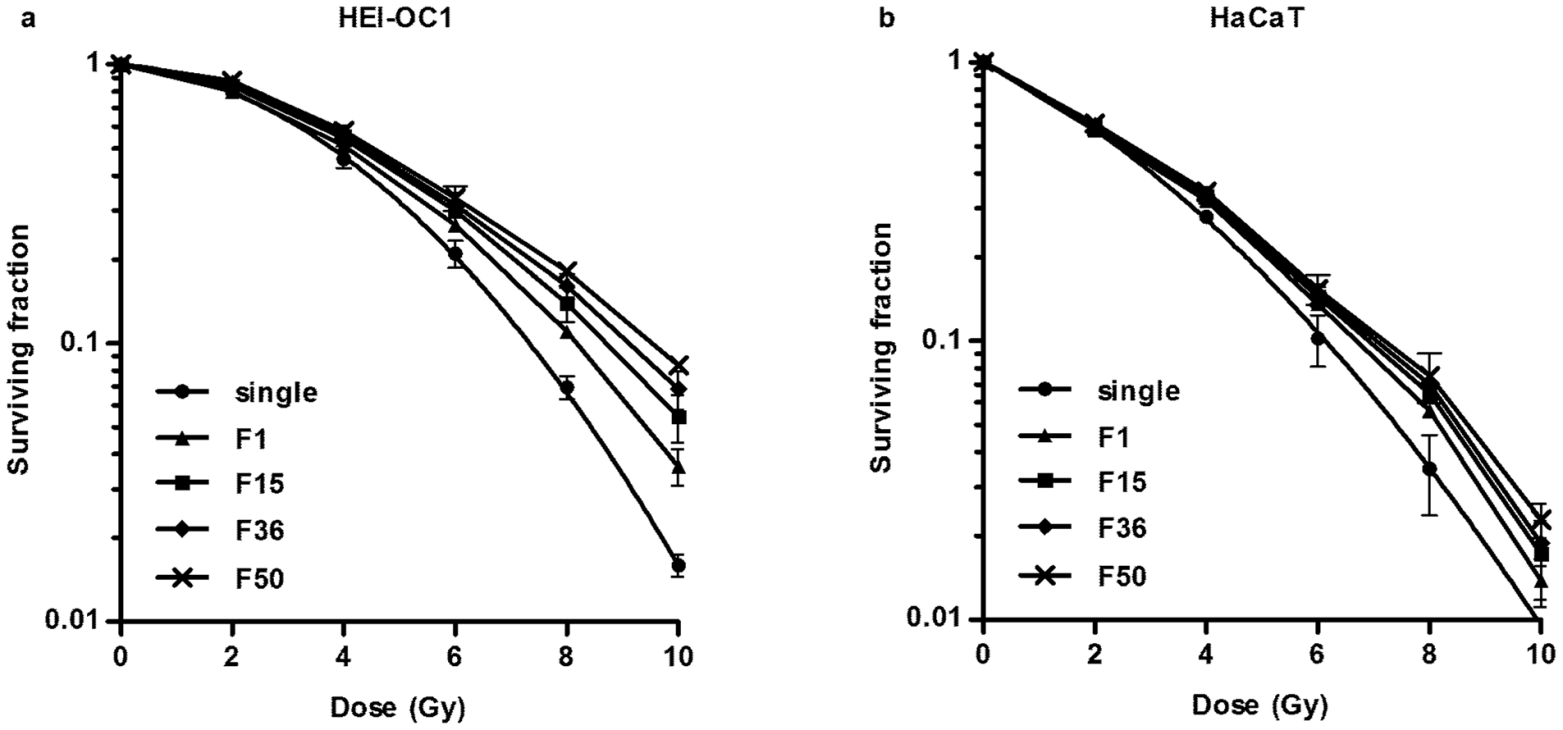
Effects of fractionated irradiation modeling EBRT and HPR on the survival of HEI-OC1 and HaCaT cells. Survival curves manually plotted based on surviving fractions of (a) HEI-OC1 and (b) HaCaT cells after fractionated irradiation with 2 Gy per fraction simulating conventional external beam radiotherapy with a FDT of 1 minute (F1) and high-precision radiotherapy with a FDT of 15 minutes (F15), 36 minutes (F36), or 50 minutes (F50) as well as survival curves plotted following the standard linear–quadratic model for single-dose irradiation (single).

**Figure 3:**
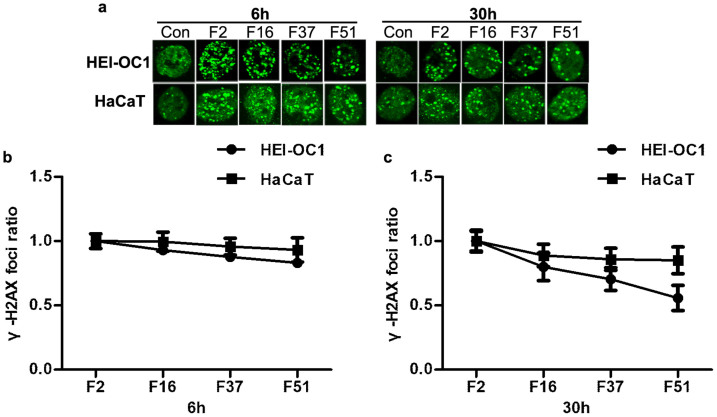
Effects of prolonged FDTs on radiation-induced γ-H2AX foci formation in HEI-OC1 and HaCaT cells. (a) γ-H2AX foci formation was analyzed using the immunofluorescence assay for HEI-OC1 and HaCaT cells at 6 h or 30 h after a single irradiation of 10 Gy with different FDTs. (b and c) The data were represented as the mean ± SEM of the ratio of γ-H2AX foci formation number after irradiation with prolonged FDTs to FDT of 2 minutes.

**Figure 4:**
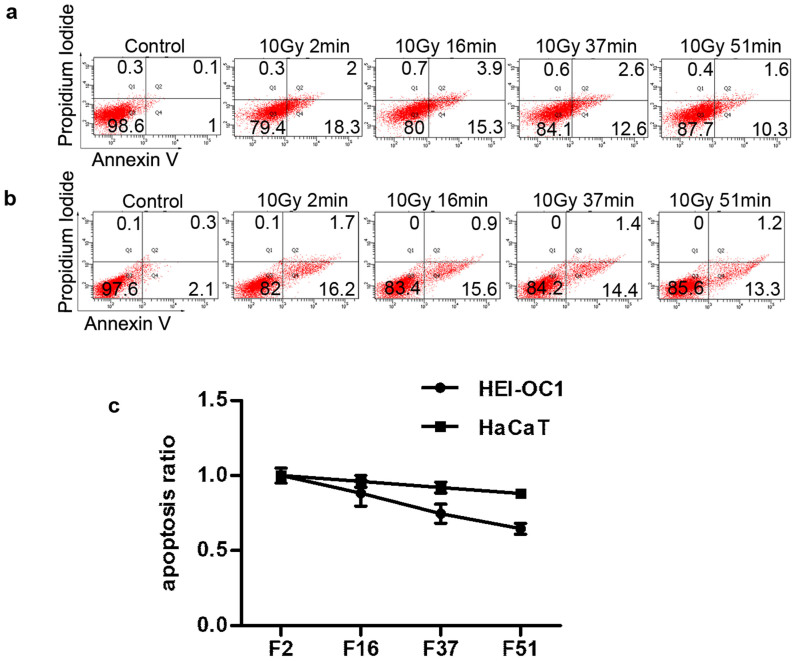
Effects of prolonged FDTs on radiation-induced apoptosis in HEI-OC1 and HaCaT cells. At 24 h after single irradiation of 10 Gy with different FDTs, apoptosis of (a) HEI-OC1 and (b) HaCaT cells was assessed using flow cytometry with an Annexin V-FITC/PI apoptosis detection kit. (c) The data are represented as the mean ± SEM of the ratio of apoptosis by irradiation with prolonged FDTs to FDT of 2 minutes.

**Figure 5:**
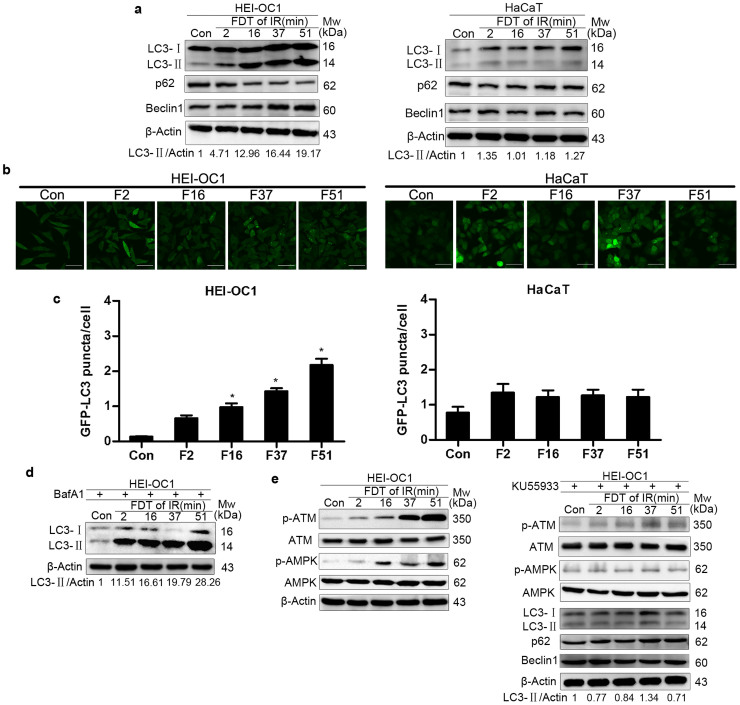
Effects of prolonged FDTs on radiation-induced autophagy. (a) Cells untreated (control) or exposed to a single irradiation of 10 Gy with a FDT of 2 minutes (F2), 16 minutes (F16), 37 minutes (F37) or 51 minutes (F51) were subjected to immunoblotting using antibodies against LC3, p62, Beclin-1 and β-actin. Representative blots of three independent experiments are shown. (b and c) HEI-OC1-GFP-LC3 and HaCaT-GFP-LC3 cells were irradiated with different FDTs and visualized using confocal microscopy. Scale bars represent 50μm. * p < 0.05, compared with FDT of 2 minutes. (d) Western blotting analysis of irradiated HEI-OC1 cells pretreated with BafA1 (100 nM). (e) Immunoblotting was employed to examine the expression of ATM, AMPK and autophagy related proteins of irradiated HEI-OC1 cells with or without pretreated with KU55933 (100 nM). Uncropped images of blots are presented in Supplementary Fig. S4 online.

**Figure 6:**
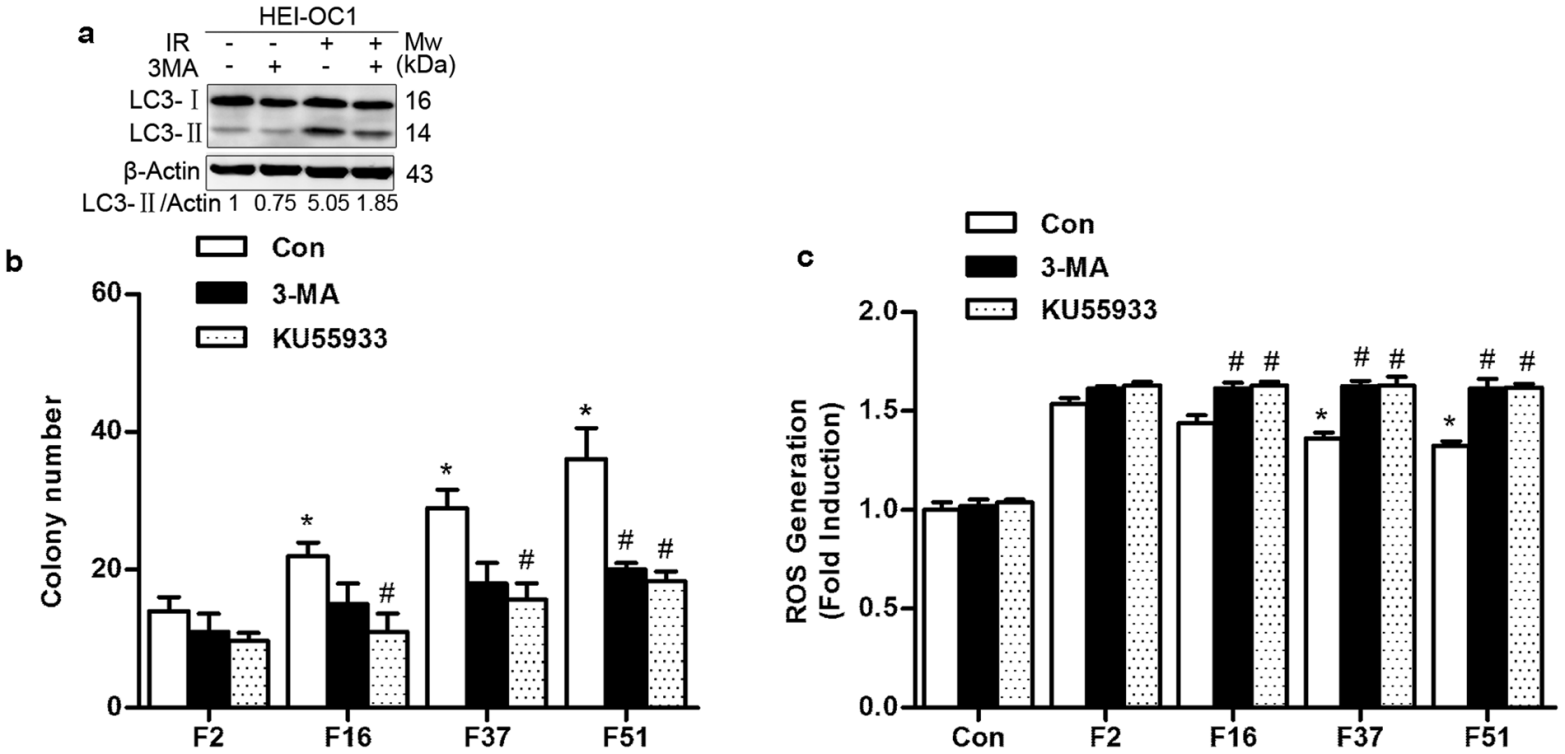
Autophagy regulated the effect of prolonged FDT protection on cell killing in HEI-OC1 cells. (a) Western blotting analysis of irradiated HEI-OC1 cells with or without pretreated with 3-MA (5 mM). (b) Survival of irradiated HEI-OC1 cells in the presence or absence of 3-MA (5 mM) or KU55933 (100 nM) was analyzed by colony formation assays. (c) The level of intracellular ROS was measured using the fluorescent probe, DCFH-DA. The presented data were obtained from three replicates and expressed as the mean ± SEM. * p < 0.05, compared with FDT of 2 minutes of control group. # p < 0.05, compared with control group of each FDT.

Irradiation was performed at room temperature using a 6-MV X-ray unit (Varian 2100C linear accelerator). Dosimetry measurements were performed by using a 0.125 cc ion-chamber (semiflex, PTW, Freiburg, Germany) on the same location in control culture media as cells. To compare the cell-killing effectiveness of irradiation-simulating conventional EBRT and HPR with different FDTs, fractionated irradiation of 0 Gy, 2 Gy × 1, 2 Gy × 2, 2 Gy × 3, 2 Gy × 4, and 2 Gy × 5 was given with one fraction per day, similar to the clinical dose–time–fractionation pattern. As shown in Fig. 1, in irradiation simulating conventional EBRT, the fraction dose of 2 Gy was delivered continuously. In irradiation simulating HPR with different FDTs, each fraction dose of 2 Gy was given in 8 equal subfractions at a dose rate of 2 Gy/min and inter-subfraction intervals of 2, 5, and 7 minutes, such that the total FDT was 15, 36, or 50 minutes (Fig. 1a). To investigate the effect of different FDTs on cell apoptosis, DNA damage and autophagy, a dose of 10 Gy was given in 8 equal subfractions at a dose rate of 5 Gy/min; the total FDT was 2, 16, 37, or 51 minutes (Fig. 1b).

### Colony formation assays

Clonogenic survival of normal tissue cells was determined using the colony-forming assay. A total of 2,000 cells were seeded in 6-cm culture plates for HaCaT, HL-7702 and MCF-10A, or 200 wells of three 96-well culture plates for HEI-OC1. Increasing numbers of cells were seeded in 6-cm culture plates for CNE1 and CNE2. After irradiation, the cells were incubated in an undisturbed state for two weeks. Colonies in 6-cm culture plates were fixed and stained with crystal violet, and the number of colonies with more than 50 cells was scored by visual inspection. The number of colonies in the 96-well place with more than 50 cells was scored using a microscope. Dose–survival curves for single-dose irradiations were plotted according to the standard linear–quadratic model using GraphPad Prism 5.0 software (GraphPad Software, San Diego, CA), and those for fractionated irradiations with 2 Gy per fraction were manually plotted.

### Apoptosis Assay

FITC-conjugated Annexin V was used to detect the presence of apoptosis. The cells were seeded in 6-cm dishes one day prior to radiation exposure. After irradiation, the cells were incubated for 24 h and then harvested and stained using the Annexin V-FITC/PI apoptosis detection kit (Invitrogen, Inc., Carlsbad, CA, USA) according to the manufacturer's instructions. The resulting fluorescence was detected using flow cytometry.

### Western blotting analysis

Western blotting analyses were performed as previously described^25^. Blots were incubated with the indicated antibodies and visualized using an ECL detection kit (Millipore). Membrane densitometric quantification of the western blot signal intensity was performed using Image Pro Plus 6.0 software.

### Immunofluorescence microscopy

Cells were cultured on coverslips and subjected to treatments as indicated. Cells were fixed and stained with a primary antibody for anti-γ-H2AX (Abcam Inc., Cambridge, MA, USA) as previously described^26^. All matched samples were examined using laser scanning fluorescence confocal microscope (Olympus, Japan). γ-H2AX foci were quantified and averaged from ten images (× 60), which were obtained from three independent experiments.

### GFP-LC3 Assay

Cells were plated into 24 wells and transfected with GFP-LC3 using a lentiviral vector for 24 hours, and cells stably transfected with GFP-LC3 were screened for GFP-LC3 Assay. Cells were irradiated and then fixed in 4% paraformaldehyde. Fixed cells were examined under a laser scanning fluorescence confocal microscope (Olympus, Japan). Ten images (× 60) were randomly selected from three independent experiments to determine the average number of GFP-LC3 punctate dots per cell.

### Determination of ROS generation

Changes in intracellular ROS levels were determined by measuring the oxidative conversion of cells permeable to 2′, 7′-dichlorofluorescein diacetate (DCFH-DA) to fluorescent dichlorofluorescein (DCF). Cells were grown in 96-well culture plates at an optimal density of 5 × 10^3^ cells/ml with 200 μl of culture medium per well. One day after plating, the cells were exposed to irradiation and then incubated for an additional 24 h. After removing the medium, the cells in the plates were washed with PBS and incubated with DCFH-DA at 37°C for 20 min. Cells were washed with PBS and DCF fluorescence was subsequently detected at an excitation wavelength of 488 nm and an emission wavelength of 525 nm using a fluorescent microplate reader (SpectraMax M5, Sunnyvale, CA, USA).

### Statistics

Data were analyzed using SPSS version 13.0 (SPSS, Chicago, IL, USA). Differences in the surviving fractions after fractionated irradiations with different FDTs were tested using the paired two-tailed t-test. Differences in colony numbers, LC3 puncta and ROS generation after 10 Gy irradiation with different FDTs were tested using the two independent samples two-tailed t-test. The results obtained from at least three independent experiments with P < 0.05 were considered significant.

## Author Contributions

Y.Y. designed and supervised the study. Q.Y. and R.Z. performed experiments and wrote the manuscript. G.X. and S.D. analyzed the data. C.R., R.L. and G.L. contributed to the data analysis and discussion. X.L and D.H. performed the irradiation.

## Supplementary Material

Supplementary Information Supplementary Information
