# Supplementary material for: Late-responding normal tissue cells benefit from high-precision radiotherapy with prolonged fraction delivery times via enhanced autophagy
Source: Sci Rep. 2015 Mar 13;5:9119. doi: 10.1038/srep09119 (PMC4357857; doi:10.1038/srep09119)
Supplement: Supplementary Information [file srep09119-s1.pdf]

Title:

Late-responding normal tissue cells benefit from high-precision radiotherapy with prolonged fraction delivery times via enhanced autophagy

Short running title: Dose protraction effect on different normal cells

Qiwei Yao[1†], Rong Zheng[1†], Guozhu Xie[1†], Shasha Du[1], Chen Ren[1], Rong Li[1], Guixiang Liao[1], Xiaoshan Lin[1], Daokun Hu[1], Yawei Yuan[1]*

[†]Contributed equally to this work.

[1]Department of Radiation Oncology, Nanfang Hospital, Southern Medical University, Guangzhou, Guangdong 510515, P.R. China.

*Please address correspondence to: Professor Yawei Yuan, Department of Radiation Oncology, Nanfang Hospital, Southern Medical University, No. 1838 Guangzhou DaDao Bei, Guangzhou, Guangdong 510515, P.R. China. Telephone number: +862061642136. E-mail: yuanyw66@aliyun.com

Table S1. Results of t tests for surviving fractions of HEI-OC1 and HaCaT after fractionated irradiation simulating conventional EBRT and HPR with fraction delivery time of 15, 36, or 50 minutes

Fraction delivery time protocols	HEI-OC1		HaCaT	
	t	p	t	p
EBRT vs. HPR with FDT of 15 min	4.255	0.001	1.637	0.124
EBRT vs. HPR with FDT of 36 min	6.197	0.000	2.039	0.061
EBRT vs. HPR with FDT of 50 min	7.568	0.000	2.706	0.017
HPR with FDT of 15 min vs. HPR with FDT of 36 min	2.296	0.038	0.891	0.388
HPR with FDT of 15 min vs. HPR with FDT of 50 min	3.293	0.005	1.836	0.088
HPR with FDT of 36 min vs. HPR with FDT of 50 min	2.337	0.035	0.856	0.407

Abbreviations: EBRT = external beam radiotherapy; HPR = high-precision radiotherapy; FDT = fraction delivery time

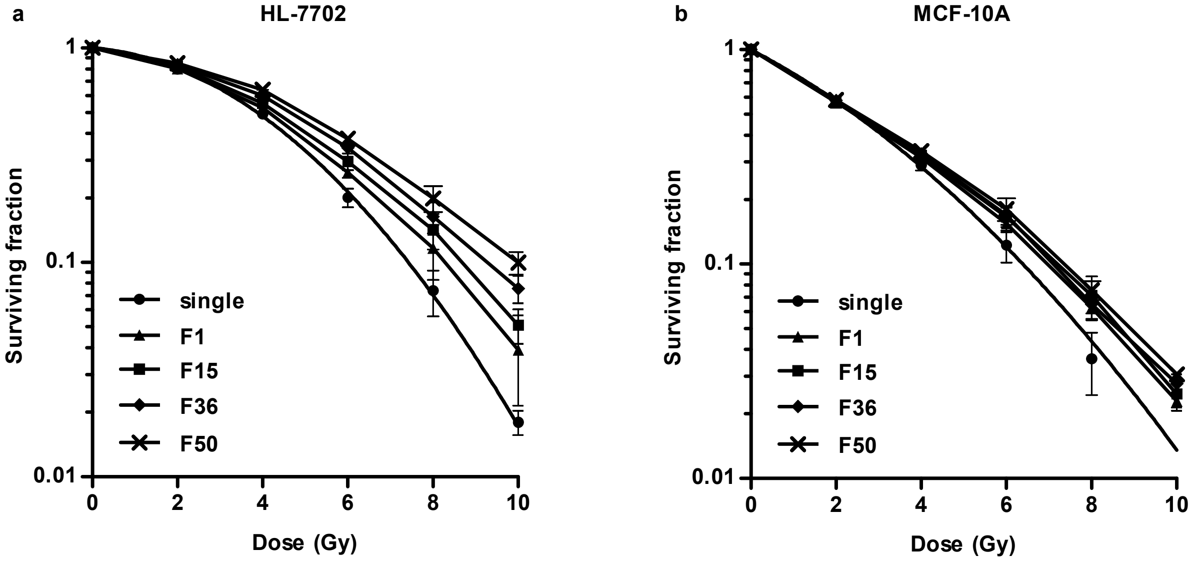

Figure S1. Effects of fractionated irradiation modeling EBRT and HPR on the survival of HL-7702 and MCF-10A cells. Survival curves manually plotted based on surviving fractions of (a) HL-7702 and (b) MCF-10A cells after fractionated irradiation with 2 Gy per fraction simulating conventional external beam radiotherapy with a FDT of 1 minute (F1) and high-precision radiotherapy with a FDT of 15 minutes (F15), 36 minutes (F36), or 50 minutes (F50) as well as survival curves plotted following the standard linear-quadratic model for single-dose irradiation (single).

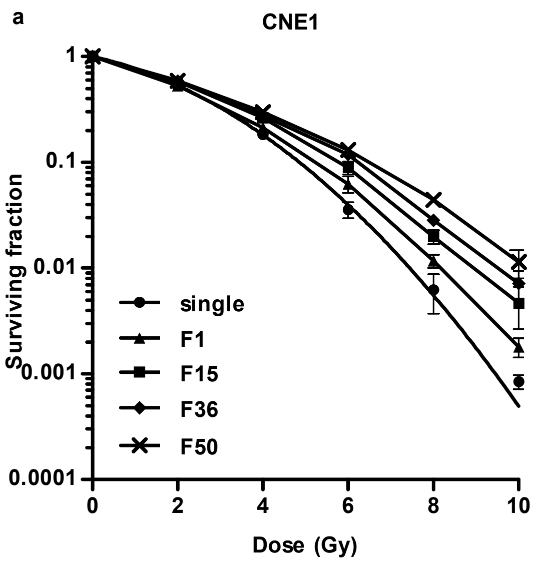

Figure S2. Effects of fractionated irradiation modeling EBRT and HPR on the survival of CNE1 and CNE2 cells. Survival curves manually plotted based on surviving fractions of (a) CNE1 and (b) CNE2 cells after fractionated irradiation with 2 Gy per fraction simulating conventional external beam radiotherapy with a FDT of 1 minute (F1) and high-precision radiotherapy with a FDT of 15 minutes (F15), 36 minutes (F36), or 50 minutes (F50) as well as survival curves plotted following the standard linear-quadratic model for single-dose irradiation (single).

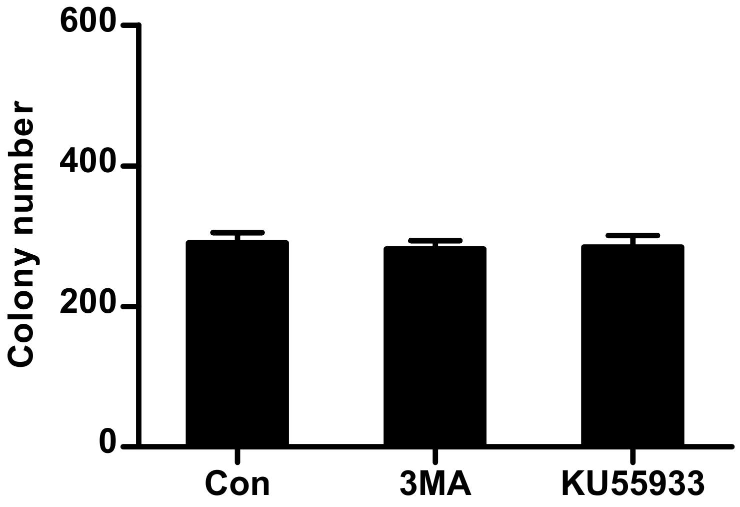

Figure S3. Survival of non-irradiated HEI-OC1 cells in the presence or absence of 3-MA (5 mM) or KU55933 (100 nM) was analyzed by colony formation assays.

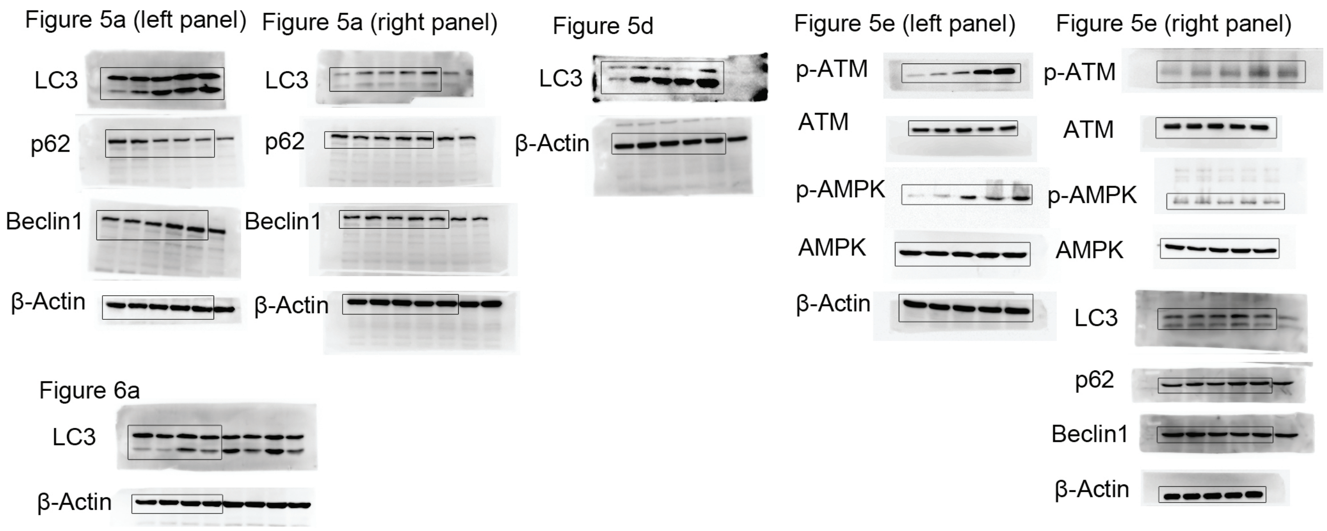

Figure S4. Uncropped images for the key experiments in the main figure.
